# Why Are Botulinum Neurotoxin-Producing Bacteria So Diverse and Botulinum Neurotoxins So Toxic?

**DOI:** 10.3390/toxins11010034

**Published:** 2019-01-11

**Authors:** Bernard Poulain, Michel R. Popoff

**Affiliations:** 1 Institut des Neurosciences Cellulaires et Intégratives, (INCI)-CNRS, UPR 3212 Strasbourg, France; poulain@inci-cnrs.unistra.fr; 2 Bacterial Toxins, Institut Pasteur, 75015 Paris, France

**Keywords:** botulinum neurotoxins, *Clostridium botulinum*, botulism, neuroexocytosis, SNARE proteins

## Abstract

Botulinum neurotoxins (BoNTs) are the most lethal toxins among all bacterial, animal, plant and chemical poisonous compounds. Although a great effort has been made to understand their mode of action, some questions are still open. Why, and for what benefit, have environmental bacteria that accidentally interact with their host engineered so diverse and so specific toxins targeting one of the most specialized physiological processes, the neuroexocytosis of higher organisms? The extreme potency of BoNT does not result from only one hyperactive step, but in contrast to other potent lethal toxins, from multi-step activity. The cumulative effects of the different steps, each having a limited effect, make BoNTs the most potent lethal toxins. This is a unique mode of evolution of a toxic compound, the high potency of which results from multiple steps driven by unknown selection pressure, targeting one of the most critical physiological process of higher organisms.

## 1. Introduction

Botulinum neurotoxins (BoNTs) are the most potent protein toxins among bacterial, animal, plant and chemical poisonous substances so far known, with a lethal parenteral dose for BoNT type A of about 0.2–0.3 ng/kg in mice and 1 ng/kg in human [1,2]. A small amount of 30 ng is sufficient to induce botulism in human adults by the oral route [3]. BoNTs are responsible for botulism, which is a rare but severe neurological disease characterized by flaccid paralysis, inhibition of secretion, and mild dysautonomia. Deciphering the poisoning process, how they disseminate in the host body, and what the different cellular steps of their mechanism of action are have revealed how highly sophisticated these nano-scale neurotropic weapons are. Several physiologically distinct bacteria produce closely related types of these deadly poisons. These pathogens are mainly environmental bacterial from soil, sediments, and occasionally the intestinal content of man and animals. Despite the fact that they have not developed a strategy to invade and survive in a vertebrate host, these bacteria have evolved to produce a potent toxin targeting specifically the neuroexocytosis machinery, which is designed to kill a host at a distance from the site where they replicate and grow. This is a very unusual property. Does this confer them some competitive advantage favoring the spreading of the neurotoxin genes among different bacterial species? The aim of this short review is to comment on the different fascinating aspects of these most potent toxins.

## 2. Diversity of Botulinum Neurotoxins and Toxin Complexes

BoNTs are produced as single-chain proteins (~150 kDa, MW). Until one decade ago, only 7 BoNT toxinotypes (A to G), defined by neutralization with corresponding specific antibodies, were recognized. Then, a chimeric BoNT type FA or HA (also called BoNT/H) was identified in a bivalent *C. botulinum* Bh strain responsible for infant botulism [4,5,6]. This discovery was followed by that of another putative novel type, called X, in a bivalent *C. botulinum* B strain, the toxicity of which remains to be determined [7]. *bont* gene sequence comparisons have permitted the identification of a large, still increasing, number of subtypes for each toxinotype (globally more than 40) [8]). For example, BoNT/A is subdivided into BoNT/A1 to BoNT/A8, BoNT/B into BoNT/B1 to BoNT/B8, BoNT/E into BoNT/E1 to BoNT/E12, and BoNT/F into BoNT/F1 to BoNT/F8 [8]. Most often, a given toxigenic strain produces only one BoNT type and subtype. However, some rare strains, called bivalent or trivalent strains, synthesize two BoNT types such as Ba, Bf, Ab, Af (the lower-case letter meaning that the corresponding type is produced in a minor amount) [8] (Table 1 and Table 2).

All the various types of BoNT are subsequently processed by endogenous or host proteases into a heavy (Hc) and a light (Lc) chain, linked together by a disulfide bond. Moreover, they are co-synthetized with one conserved associated protein, NTNH (non-toxic non-hemagglutinin protein, 150 kDa), and several hemagglutinins (HAs) or proteins of unknown properties (OrfX). At the 3D structure level, NTNH looks very similar to BoNT, except that it is devoid of neurotoxic activity. The association of a BoNT molecule with a NTNH copy confers to BoNT a long-term stability, supporting a chaperone activity for NTNH [9]. A large variety of multimeric complexes (referred to as “botulinum toxin complexes” or “progenitor toxins”) can be formed. Each of them contains only one copy of BoNT, which can be released upon the exposure of botulinum complex to neutral or mildly basic pH [10].

## 3. Diversity of BoNT-Producing Bacteria: *Clostridia*, et al.

To date, the production of BoNTs has been reported in Gram-positive, sporulating anaerobic bacteria belonging to the *Clostridium* genus. This is not surprising because among more than 200 *Clostridium* species, 15 produce very potent toxins, which are responsible for severe diseases in humans and animals. Since BoNT-producing bacteria share the same phenotype consisting of producing flaccid paralyzing neurotoxins, most of the neurotoxigenic clostridia have been named *Clostridium botulinum*, without consideration of their physiological heterogeneity. Based on phenotypic markers (e.g., proteolysis, lipolysis, carbohydrate utilization), 16 rRNA gene sequences, and a whole-genome sequence comparison, neurotoxigenic *Clostridia*-producing BoNT belongs to at least six groups (I to VI) corresponding to distinct bacterial species. The so-called *C. botulinum* strains of group I produce highly thermoresistant spores and grow between 10 °C and 37 °C, whereas strains of group II are non-proteolytic, have moderate thermoresistant spores, and grow and produce toxins at temperatures as low as 3.5 °C. *C. botulinum* C and D, mainly responsible for animal botulism, preferentially grow at higher temperature s(37–40 °C) and are referred to as group III. *C. argentinense*, which produces BoNT/G, is the prototype of group IV. In addition, several other, albeit atypical, strains of *Clostridium* species are neurotoxigenic: certain *C. baratii* strains produce BoNT/F (referred to as BoNT/F7) and some *C. butyricum* strains synthesize BoNT/E (referred as BoNT/E4 and E5). They are assigned to groups V and VI, respectively, and show phenotypic properties related to those of the corresponding *Clostridium* species type strains [11,12]. Moreover, the neurotoxigenic strains of each group or species display a genetic variability. For example, multilocus sequence typing defines several phylogenetic clusters in each group [13,14,15,16,17,18].

Moreover, the screening of *bont* gene sequences in available genomic sequences in databases have revealed that *bont* sequences are not restricted to clostridia. Non-clostridial *bont* homologs have been identified in other anaerobes and aerobes. *bont*-like gene clusters have been identified in *Weisella oryzae* (referred as BoNT/I or BoNT/Wo), which is a Gram-positive, non-spore-forming, facultative anaerobe from fermented rice [19], and in *Chryseobacterium piperi* (referred as BoNT/Cp1), which is a Gram-negative, non-spore-forming, strictly aerobic bacterium from the environment [20,21]. In addition, an *Enterococcus faecium* strain isolated from cow has been found containing a *bont*-related gene in an OrfX cluster that encodes for BoNT/En. This BoNT, also called BoNT/J, shares a 38.7% identity with BoNT/X and is more distantly related (23–25% identity) to the other BoNTs and tetanus neurotoxin (TeNT) [22,23]. The production of functional BoNTs by these non-clostridial microorganisms has not yet been reported. However, the question of whether BoNTs should still be considered as only clostridial toxins is now open.

## 4. Though Distinct, the BoNTs Cause Similar Diseases

Intoxication with one of the various BoNT types or subtypes causes a unique severe neurological disease: botulism [24,25]. Overall, the disease results from the inhibition of cholinergic neurotransmission in the peripheral part of the nervous system. In mammals, botulism is characterized by mild dysautonomia (including the inhibition of gland secretion) and prominent muscle fatigue or flaccid paralysis. In the most severe forms, respiratory distress occurs and may be fatal without treatment. The clinical manifestations of botulism may vary in a subtle manner within the different toxinotypes and even subtypes. For instance, dysautonomia is more marked after poisoning with BoNT/B than with BoNT/A [24,25]; type F botulism is also a severe form of botulism but with a shorter duration compared to type A botulism [26].

Human foodborne botulism, subsequent to the ingestion of preformed BoNT in food, is common in many countries, such as in Europe. It is mainly caused by types A, B, E and more rarely F. Botulism can also result from *C. botulinum* intestinal colonization and subsequent local BoNT production in human infants (infant botulism is the main form in the US) and more rarely in adults with immature/altered microbiota. In addition, rarer forms have been reported such as after the colonization of a necrotizing wound, botulism by inhalation, and iatrogenic botulism. Large outbreaks of animal botulism have been reported in mammals (e.g., cattle, minks, ferrets, foxes), birds (e.g., waterfowl) due to intoxication by types C, D, dominantly. Fishes are also susceptible to the disease (types E, B, and C) [12,25,27,28,29,30].

## 5. BoNTs Are Designed to Kill a Distant Host

**Figure 1:**
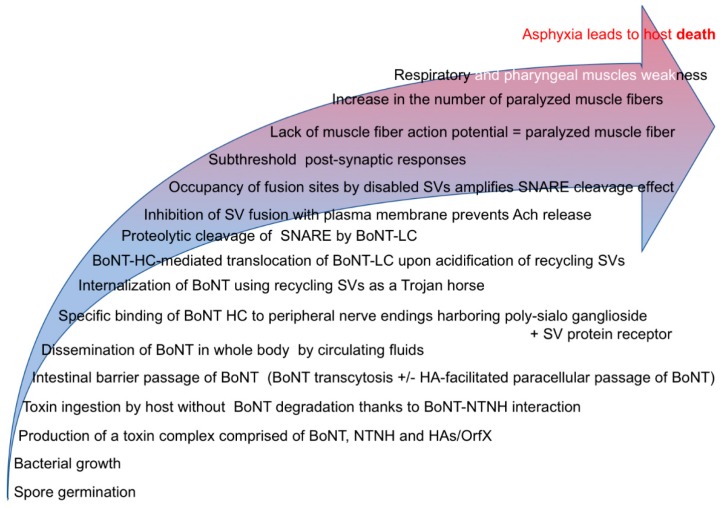
Schematic summary of the BoNT activity steps.

In most of the infectious bacterial diseases, bacteria colonize the host organism they affect. With the BoNT-producing bacteria, the situation is very different (Figure 1). Indeed, they produce a toxin that affects their final targets (i.e., the nerve terminals) in a host living at a distance, spatially and chronologically, from the site where the bacteria replicate. Foodborne botulism is due to the ingestion of a preformed toxin contained in inadequately preserved food (human), poorly prepared silage (cattle) or decaying organic matter or carcasses (many animal species). Live maggots feeding on carcasses can accumulate enough toxin to cause foodborne botulism in their vertebrate predators, without being poisoned by BoNTs [31,32]. Remarkably, host intoxication can occur a long time, days up to years, after the neurotoxin has been synthetized and even after bacterial death, such as in matrices preventing *C. botulinum* sporulation, for example, in some canned foods [33,34]. This is possible thanks to the exceptional stability of the NTNH–BoNT complexes to acidic pH and to proteolytic degradation [9]. Thus, BoNT can pass through the digestive tract (i.e., through the very acidic and protease-rich stomach as well as the proteolytic intestinal content) in a non-inactivated form. Thereby, BoNTs can cross without damaging several physiological barriers such as the stomach and intestinal epithelium [35,36]. During foodborne and infant botulism, BoNT molecules undergo passage through the stomach and/or intestinal epithelium [35,36], either by transcytosis through intestinal epithelial cells [37] or through the paracellular way thanks to the interaction of HAs with E-cadherin and subsequent disruption of intercellular junctions [35,38]. Dissemination of BoNTs in the body through blood and lymph circulation allows BoNT molecules to reach the peripheral nerve endings [35,36]. BoNT cannot gain direct access to the central nervous system (CNS) across the blood–brain barrier [36,37,39,40]. However, similarly to TeNT, very minute amounts of BoNT can enter the CNS using a transcytosis mechanism in neurons via the retrograde pathway [41]. Although BoNT receptors mediating the toxin entry into target neuronal cells have been extensively analyzed (review in [42]), those driving BoNT sorting into retrograde transport vesicles remain to be defined.

## 6. BoNTs Are Designed to Selectively Recognize Nerve Terminals and Exploit Synaptic Vesicle Recycling as a Trojan Horse to Enter into Them

What confers the exquisite neurotropic specificity of the neurotoxins? The C-terminus portion of Hc contains domains mediating binding to target nerve terminals through an interaction with double membrane receptors comprised of a poly-sialo-ganglioside acting as a low-affinity receptor in the vertebrates, enriched in the outer leaflet of plasma membranes of neuron nerve endings, and a high-affinity receptor consisting of a glycoprotein protein (review in [42,43]). It is noteworthy that the binding affinity of BoNTs for their receptors is in the same range as those of other potent lethal bacterial toxins (Table 3). Thus, BoNTs are neurotoxins that recognize specific receptors on target neuronal cells, but they have not developed an exceptional binding affinity to interact with them. Overall, the BoNTs prefer complex gangliosides rather than a simple one. They have a considerably higher affinity for the b series gangliosides such as GT1b/GD1b, than with GM1 or GM3; binding to GD1a is high as well (review in [40,42]). The preference for gangliosides varies with the toxinotype. For instance, the affinity of BoNT/A is high for GT1b > GD1b >> GM1 [44]. BoNT/B affinity is high for GT1b and GD1a and much lower for GD1b and GM1 [45] and that of BoNT/F is higher for GT1b and GD1a than for GM3 and very low for GD1b or GM1a [46]. Of interest, the dominant forms of gangliosides in neurons are the complex forms including GM1, GD1a, GD1b, and GT1b, which are more enriched (1 to 2 order of magnitude higher) in nerve cell membranes than in other cell types [47,48], thereby explaining BoNT tropism for the nervous system.

Depending on the BoNT toxinotype, the protein receptor is one of the vesicle membrane proteins: N-linked glycan-SV2 (-A, -B, or -C) or synaptotagmin (−1 or −2). This interaction with a dual receptor on cell membrane avoids the binding of BoNT to non-receptive cells and facilitates specific trapping from the extracellular space and concentration into neuron endings. The two binding sites for gangliosides and glycoprotein, respectively, have been characterized on the HcC-terminal domain [42,43]. Interestingly, BoNT interaction with SV2 isoforms requires both the recognition of a protein domain and a glycan N-lined to SV2 [49,50]. The recognition of N-linked glycan in addition to the protein part of a receptor increases the specificity of the host–pathogen or bacterial toxin interaction with target cells [51]. BoNT/B, D/C, and G, which interact with synaptotagmin, use an additional interaction with the lipid membrane via a hydrophobic loop located in their Hc between the ganglioside and synaptotagmin binding sites [52]. Moreover, both co-receptors (poly-ganglioside + protein) have to be co-localized into the same membrane microdomain [53]. Thus, during evolution, Hc looks to be tuned for maximizing the interaction of BoNT with neuron endings, thereby facilitating its ensuing neuronal uptake. Indeed, SV2 and synaptotagmin are integral proteins of synaptic vesicle membrane whose luminal domain is exposed onto the nerve-ending surface upon the collapse of synaptic vesicles with the plasma membrane during neurotransmitter exocytosis. This allows the trapping of BoNT inside recycling synaptic vesicles, the acidification of which triggers the translocation of Lc into the cytosol, and at the same time, the disulfide bridge linking Lc to Hc is reduced, making Lc free in the cytosol and unmasking its catalytic cleft [42,54,55,56,57]. Hence, recycling synaptic vesicles act as the main Trojan horse (Figure 1) that introduces the neurotoxins into the nerve terminals at only a few tens or even hundreds of nanometers from the site where their final molecular targets, the soluble N-ethylmaleimide-sensitive-factor attachment protein receptor (SNAREs), are concentrated.

## 7. BoNTs Are Not Super-Enzymes but Their Effect Is Amplified at Many Steps of Their Action

Lc is a Zn-dependent metalloprotease [67]. In the cytosol, depending on the BoNT toxinotype, Lc specifically cleaves only one of the three SNARE proteins (either synaptosomal nerve-associated protein 25 (SNAP-25), vesicle-associated membrane protein (VAMP)/synaptobrevin, or syntaxin) (Table 1). The high proteolytic specificity of Lc for a unique substrate results from a pairing of one to two SNARE motifs (in addition to the cleavage site) with exosites present in the Lc catalytic cleft [68,69,70]. Given the key role for the SNAREs in mediating the fusion of synaptic vesicles with plasma membrane exocytosis, their cleavage results in a blockade of Ca^2+^-dependent exocytosis of neurotransmitters (Figure 1).

Not only BoNTs inhibit neurotransmission, but they also do it over a long term. Indeed, whereas the lifespan of these neurotoxins in extracellular media is in the range of several days, this is not the case when they are intra-neuronal. Here, their lifespan is in the range of several weeks to months (reviewed in [71]). The longest-acting one is BoNT/A Lc. Its interaction with a cytosolic des-ubiquitin ligase prevents its ubiquitinylation and ensuing entry into the proteasome degradation pathway [72,73,74]. This allows for maintaining the inhibition of exocytosis for months, despite the rapid re-synthesis of the cleaved SNAREs. However, the lifetime of Lc in neuronal cells is probably not the only factor involved in the duration of BoNT effects. Indeed, SNARE complexes likely adopt a radial arrangement, and this supports the idea that SNAP25 cleaved by BoNT/A does not impair the SNARE complex assembly but acts as dominant negative SNARE oligomer that can have a long-duration inhibitory effect on neuroexocytosis machinery [75,76].

As mentioned above, BoNTs undergo a long journey between the distant site of their production (mostly in food or intestine) and the nerve endings where they act (Figure 1). Along this journey, they pass through several physiological barriers at the price of large dilution in body fluids, so that only tiny amounts reach nerve terminals (far below picomolar concentrations during the disease). Therefore, with regard to their very high lethality (a range of 100 million mice LD50/mg neurotoxins; LD ~0.2 ng/kg in case of BoNT/A), one would expect their Lc to be a super protease. However, this is not the case. Their enzymatic kinetic parameters, as investigated with BoNT/A Lc and its substrate SNAP25, revealed an enzymatic performance which is far from exceptional. The number of SNAP25 molecules cleaved by a BoNT/A Lc molecules per second (kcat) is rather low (k_cat_ = 17.1 s^−1^ [77,78], k_cat_ = 0.51 mn^−1^ (0.0085 s^−1^) [79]). This is slightly lower than other bacterial proteases (k_cat_ of thermolysin-like zinc-dependent protease of *Bacillus stearothermophilus* = 180 s^−1^) [80]. Why are BoNTs so deadly? It turns out that their incredible lethality results from a unique combination of two factors. First of all, the neurotoxins attack our Achilles’ heel: the system of communication between neurons and essential effectors such as muscles and glands, without which life cannot occur. Second, many steps optimize or even amplify their deleterious action (Figure 1):

(i) The chaperoning of BoNT by NTNH minimizes acidic pH and protease degradation upon passing through the upper digestive tract;
(ii) Receptor-mediated transcytosis and/or HA-dependent paracellular passage allows the bypassing of physiological barriers (intestinal barrier or endothelial barrier);
(iii) Specific receptors on neuronal cells trap and concentrate the toxin molecules on target cells avoiding diffusion and dilution in non-productive host compartments;
(iv) Receptor-mediated internalization by recycling vesicles optimizes neurotoxin uptake at the precise site where their molecular targets (the SNAREs) are accumulated;
(v) Nerve endings contain hundreds (most central synapses) up to several tens of thousands (motoneuron) of synaptic vesicles. Their fusion with a plasma membrane can occur only in very specialized regions (i.e., release sites) of the plasma membrane called active zones, the number of which is limited at each nerve ending. For a fusion event, a ring of several SNARE complexes should be formed at the interface of a given synaptic vesicle and plasma membrane at the release site [81]. Following cleavage by BoNT, SNAREs can form non-productive complexes. Therefore, synaptic vesicles can continue docking on release sites but do not fuse due to the presence of one or a few unproductive SNARE complexes in the ring [40,81]. Since these vesicles cannot fuse nor be retrieved, the number of release sites able to experience exocytosis decreases, as demonstrated after the cleavage of VAMP/synaptobrevin [82]. Thus, the cleavage of a small proportion of the SNAREs is sufficient to silence synaptic neurotransmission [40,81];
(vi) The long duration of the Lc of some BoNT types such as BoNT/A, which is the most potent BoNT, inside the target cells and the long duration of activity;
(vii) At the neuromuscular junction, there is no need for a complete blockade of exocytosis to get complete paralysis [83]. As soon as the number of synaptic vesicles fusing with plasma membrane in response to motor command is too low to induce subthreshold post-synaptic responses, muscle fiber contraction does not occur and muscle contraction weakens;
(viii) Asphyxia and subsequent death do not need the complete paralysis of the diaphragm and pharyngeal muscles. It occurs when muscle weakness is sufficient not to allow enough gas exchange (i.e., a vital capacity below 15 mL/kg body weight in humans) as reported for peripheral neuropathies [84]. This may explain why the lethal dose of BoNT/A in mice (25 g) by the intraperitoneal route is 3.7 pg [85] or 7 pg for highly purified recombinant toxin [86], whereas the ex-vivo nerve-hemidiaphragm assay requires 10 to 20 more toxin molecule numbers [87].

Overall, BoNTs are not super enzymatically active but super efficient (Figure 1). Their very high potency results from a unique combination of in vivo steps, each with a limited incremental effect, the accumulation of which confers to this non-cytotoxic toxin the ability to kill large organisms. This situation is unique among bacterial toxins. The other bacterial toxins that display high lethal toxicity just below the BoNTs are diphtheria toxin, *Clostridium perfringens* epsilon toxin and *Clostridium sordellii* lethal toxin [88]. In contrast to BoNTs, they are highly cytotoxic for their target cells, and this critical step is responsible for their pathological effects [89,90,91,92,93].

## 8. What about the BoNT Origin?

A large number of distinct bacteria share the same property of producing a BoNT, albeit of diverse types or subtypes. This raises the question of the origin of the BoNTs. The high level of amino acid sequences and the structural identity of all the BoNTs types and subtypes as well as NTNH proteins associated to BoNTs strongly support the possibility that they derive from a common ancestor gene. What are the gene spreading mechanisms involved in making so many different bacterial strains produce BoNTs or display *bont*-related genes? *bont* genes and those encoding non-toxic associated proteins (NTNH, HAs, or OrfX) are localized in a locus (botulinum locus) which is flanked by insertion sequences and is located in various DNA structures (chromosome, plasmid, phage, transposon or transposon-like DNA elements). Such a localization of DNA mobile elements, notably plasmid and phages, accounts for horizontal gene transfer between various clostridial strains [94,95,96], and possibly also between clostridia and other bacterial species. For instance, the gene of the novel BoNT/En (i.e., BoNT/J) is located in the *E. faecium* conjugative plasmid possibly acquired from a *Clostridium* strain [23]. In most of the *C. botulinum* B strains, the botulinum locus is located in plasmids and shows a high genetic diversity even inside each subtype [97]. In addition, most of the clostridial bivalent strains include a botulinum locus type B, suggesting that these strains are highly receptive to the acquisition/transfer of mobile elements such as plasmids and are highly susceptible to DNA modifications [95,98,99]. *C. tetani* produces a TeNT that is closely related to BoNT/B and shares with it the same cleavage site in its SNARE target [67]. Similar to the *bont* B gene, the *tent* gene is also located on a large-sized plasmid, and BoNT/B shares the highest level of its amino acid identity with TeNT. However, *tent* is not associated with non-toxic protein encoding genes. Therefore, the question of whether BoNT/B results from genetic transfer and the subsequent modification of *tent* from *C. tetani*, or vice-versa, is open. Interestingly, the *ntnh* gene is conserved in all BoNT-producing clostridia and is located just upstream of the *bont* gene with which it forms an operon, supporting the idea that *bont* and *ntnh* result from duplication of a common ancestor gene. Indeed, NTNH retains the same size as BoNT, and both NTNH and BoNTs are structurally related [9,39]. Is there a common ancestor of clostridial neurotoxin genes with duplication in *bont* and *ntnh* genes in *C. botulinum* in contrast to the single *tent* gene in *C. tetani*? It has been hypothesized that the clostridial neurotoxins have arisen from a viral protease fused to transmembrane and receptor domains [100]. However, the mode of genetic transfer between virus and clostridia is hypothetical. *C. botulinum* C and D contain phages harboring *bont*, but these phages share no significant homology with other phages or viruses [101].

## 9. Distribution of BoNT-Producing Bacteria

Until now, the established BoNT-producer has been clostridia. The usual habitat of clostridia is an anaerobic environment: soil, dust, sediment, cadavers, manure, and, depending on the species, the intestinal content of healthy animals (mammals such as, pigs, birds, and fishes). Although *C. botulinum* strains are widely distributed in the environment, there still exist geographical variations in the prevalence of certain toxinotypes. Type A and B strains are found in soils that are poor in organic matter, and more rarely in aquatic sediment. Overall, *C. botulinum* type A is predominant in the western part of the United States (west of the Missouri and Mississippi rivers), in soil that is neutral to alkaline (average pH 7.5) with a lower than average organic content. In contrast, type B prevails largely in the eastern part of the United States, and central and western Europe. B strains are recovered in slightly more acidic soil and sediments (average pH 6.25) with a higher level of organic matter content, and mainly in cultivated soils (pasture, fields) [102,103,104]. Group II *C. botulinum* strains (*C. botulinum* E, non-proteolytic *C. botulinum* B and F) can grow and produce toxin at low temperatures. Therefore, *C. botulinum* E is predominant in the northern part of Europe (Scandinavia, Finland), America (Canada, Alaska) and Asia [103] whereas *C. botulinum* B from group I and unexpectedly also from group II are more prevalent in warmer areas. Moreover, *C. botulinum* B is a frequent inhabitant of the digestive tract of healthy pigs while *C. botulinum* E is often found in the intestinal content of fish and aquatic animals living in northern countries [103,105,106]. Thus, BoNT-producing clostridia show certain distinct environmental distributions that reflect their different physiological properties better than the production of different BoNT types. The distribution of the non-toxic *C. botulinum* counterparts has not been thoroughly investigated. It is noteworthy that clostridia are widespread in the environment. For example, clostridia, and notably *C. butyricum,* are one of the most abundant bacterial groups in lake sediments and sludge [107,108]. Based on their physiological properties, such as their tolerance/sensitivity to oxygen, the requirement of an appropriate pH, temperature, substrate for growth, and spore production/germination, the repartition of *Clostridium* species in the environment is heterogeneous. Saccharolytic clostridia such as *C. butyricum* and *C. baratii* preferentially grow in carbohydrate-rich environments, notably in decomposing vegetables and fruits, whereas proteolytic and gelatinolytic *Clostridium* including toxigenic and non-toxigenic *C. botulinum* strains that poorly sporulate are mainly found in animal cadavers or soil/sediments rich in organic material [12]. The basis of the adaptation of metabolic pathways to particular substrates and/or to host defenses by the distinct *C. botulinum* types and subtypes remain to be elucidated.

## 10. Why So Potent, and for What Purpose?

A current idea is that the production of potent toxins able to kill specific animal hosts might facilitate the survival and dissemination of BoNT-producing strains in the environment by providing appropriate substrates from animal cadavers [31,32]. Is this, overall, the case with neurotoxigenic clostridia? Apparently not: the non-toxigenic strain derivatives by the loss of *bont* genes (loss of phage, plasmid, and mobile DNA elements, for example) can grow and sporulate as well as their neurotoxigenic counterparts. In addition, the toxigenic strains closely related to non-toxigenic clostridial species are widespread in the environment, further arguing that toxigenicity is not a prerequisite of survival in the environment [97,98]. The most striking example is *Clostridium sporogenes*, which is commonly considered as a non-toxic counterpart of group I *C. botulinum* strains. The genomic analysis of *C. sporogenes* shows that most strains, albeit highly related (93% average nucleotide identity) to *C. botulinum* group I strains, contain specific clade-genetic signatures and constitute a distinct bacterial species than *C. botulinum*. However, some *C. sporogenes* strains have lost these signatures and are phylogenetically clustered with *C. botulinum* group I strains [97,109]. Horizontal *bont* gene transfer has been demonstrated between strains from *C. botulinum* group I and *C. sporogenes*, further supporting the high genetic relatedness between them [96]. *C. sporogenes* is a frequent inhabitant of the environment, notably in milk, milk products, and canned foods [110,111], and is widely used as a *C. botulinum* surrogate in testing commercial food processing procedures [112,113]. This again further supports the idea that *bont* does not confer a specific advantage in *Clostridium*’s survival and spread in the environment. Therefore, what is the evolutionary pressure driving *bont* gene-spreading in a number of bacterial species (as mentioned above)? Moreover, we face another unusual situation: BoNT-producing bacteria live in an anaerobic environment and their toxins act on very distant hosts living in an aerobic environment. Even in the case of wound botulism [25], the anaerobic environment in which they grow (necrotic abscess) is not due to BoNT action but to the favorable conditions of necrotic tissue similar to those that can be found in the natural environment. The intestinal content is also an anaerobic environment, but the physiological microbiota is not favorable to the growth of these environmental bacteria [32]. It is worthwhile to note that the BoNTs responsible for the most frequent forms of foodborne botulism in human and cattle are produced in preserved food (type A and B for humans) and silage (type C, D for cattle), which are recent human artefacts, which cannot have been anticipated by evolution.

Are vertebrates the only possible hosts? The SNAREs are evolutionarily highly conserved among all eukaryotes [114]. However, the SNAREs harboring the cleavage sites attacked by BoNT or the closely-related TeNT look to appear with the nervous system of the metazoans, which exploit the Ca^2+^-regulated exocytosis of neurotransmitters, mediated by a specific subset of SNAREs and using synaptotagmin as a Ca^2+^-sensor. Indeed, SNAREs susceptible to cleavage by BoNT or TeNT are expressed in the neurons and endocrine cells of many invertebrate and vertebrate phylla. Intracellular neurotoxin or Lc expression bypassing the limiting membrane steps leads to exocytosis inhibition in Echinoidea (as sea urchin), Annelida (as the leech), Mollusca (as *Aplysia*, squid), Arthropoda (as crayfish or the fruit fly *Drosophila*), and in almost all the vertebrates (fish, birds, mammals) [40,81]. Therefore, all metazoans are potential sensitive hosts for BoNTs. However, harboring a cleavable SNARE is not sufficient for designing a potential host. Indeed, invertebrates look not to be susceptible to botulism [31,115,116] and the impairment of neurotransmission in them needs a very high extracellular concentration of BoNT (10 nM in *A. californica*) [117]. Indeed, a key limiting step in the poisoning mechanisms is receptor-mediated internalization. Although the protein receptors of some BoNTs, such as synaptotagmins (−1, −2), are well conserved during evolution; this is not the case for N-linked glycan SV2 isoforms that are lacking in invertebrates (although vertebrates and invertebrates shares the non-glycosylated SV2-related-protein SVOP [118]). Moreover, invertebrates, except echinoderms, do not synthetize the gangliosides [48] that increase BoNT’s binding affinity to neurons. Hence, the victims/hosts of prototypic BoNTs in natural conditions seem to be members of the vertebrata sub-phylum and not invertebrates. However, it is conceivable that, during evolution, the binding domain in BoNT Hc may have evolved to facilitate the exploitation of other membrane receptors, allowing these modified toxins to attack the invertebrate nervous system. If so, what would be the clinical manifestations of the disease in invertebrates?

It is difficult to conceive of what kind of evolutionary pressure pushed the clostridia to develop such sophisticated neurotropic weapons, with the ultimate ‘purpose’ of killing animals and moreover at a distance from the bacterial multiplication site. Is this to create, from time to time, a large anaerobic fermentor [32]? Perhaps we are barking up the wrong tree: indeed, the production of BoNT might be a “quality” independent from bacterial survival, as recently proposed [119], which does not confer any advantage to the bacteria. Thereby, botulism might be the result of accidental encounters between unfortunate receptive hosts and neurotoxigenic environmental bacteria rather than a beneficial and prerequisite interaction for the pathogen. However, this type of accident looks to be highly frequent enough to have exerted some evolutionary pressure on the hosts: SNARE mutations conferring resistance to cleavage concentrates in certain highly exposed animal species (rat and chicken VAMP-2 [40,67,81]. Does the genetic diversity of clostridia strains and corresponding BoNT variants involved in the various forms of botulism (foodborne, infant, intestinal, and wound botulism) reflect the fact that the strains are found in the environment where the host is living rather than a pathogen adaptation to a specific host [18,33,120,121]? Since *bont*-related genes have been recently identified in other bacteria than *Clostridia* (see above Section 3), it is conceivable that BoNT-ancestor related toxins and corresponding hosts will be discovered in the future, shedding light on the evolutionary mechanisms pushing many bacteria to adopt such a potent toxin arsenal.

## 11. Concluding Remarks

It is to our discredit that we have failed to answer the introductory questions: the mystery still remains. Indeed, what the protease ancestor gene could be that gives rise to the unique situations that different bacteria share closely related toxins remains to be elucidated. Even more enigmatic is why and how environmental bacteria have acquired such sophisticated and active toxins characterized by extreme specificity towards highly specialized proteins from the metazoan neural machinery of neuroexocytosis. What is the advantage for them to produce a lethal toxin that can kill a host at a distance from the bacterial replication site? Since the identification of botulism as a natural poison-caused disease by Justinus Kerner [122,123], two centuries of hard work have been necessary to understand botulism’s mechanisms at the molecular level. The natural history of the BoNTs and their producing bacteria is still in progress. From the suspicion of a neurotoxic compound in some contaminated foods responsible for a severe neurological disease to the characterization of BoNT activity in the neuroexocytosis process at the molecular and structural levels, a major breakthrough has been reached. However, a complete understanding of these toxins, which show a great diversity and use a sophisticated multi-step activity to become the most potent toxins, remains to be further developed.

## Figures and Tables

**Table 1:** Botulinum neurotoxin (BoNT) types, subtypes and producer organisms.

Botulinum Toxin Type	BoNT/A	BoNT/B		BoNT/E	BoNT/F			BoNT/E	BoNT/C	BoNT/D	BoNT/G	BoNT/H	BoNT/Ba BoNT/Bf BoNT/Ab BoNT/Af BoNT/A(B) BoNT/A2F4F5
Subtypes	A1, A2, A3, A4, A5, A6, A7, A8	B1, B2, B3, B5, B6, B7, B8	B4	E1, E2, E3, E6, E7, E8, E9, E10, E11, E12	F6	F2, F2, F3, F4, F5, F8	F7	E4, E5	C/D, D/C		G	H or F/A or H/A	
Enzymatic substrate (cleavage site)	SNAP25 (QR)	VAMP1, 2, 3 (QF)		SNAP25 (RI)	VAMP1, 2, 3 (QK) F5: VAMP2 (LE)		VAMP2 (QK)	SNAP25 (RI)	SNAP25 (RA) Syntaxin (KA)	VAMP1, 2, 3 (KL)	VAMP1, 2, 3 (AA)	VAMP1, 2, 3 (LE)	
Neurotoxin-producing bacteria	*C. botulinum*		*C. botulinum*			*C. botulinum*	*C. baratii*	*C. butyricum*	*C. botulinum*		*C. argentinense*	*C. botulinum* bivalent/trivalent strains	
Group	Group I		Group II	Group II	Group II	Group I	Group V	Group VI	Group III		Group IV	Group I	
Botulism	Human Occasionally animal						Human Animal not reported		Animal Very rare in human		No natural case reported	Human	Human

**Table 2:** Putative novel botulinum neurotoxin (BoNT) types and producer organisms.

Botulinum Toxin Type	BoNT/X	BoNT/I or BoNT/Wo	BoNT/J or eBoNT/J or BoNT/En	Cp1 Toxin (BoNT Homolog)
Subtypes	Bivalent BoNT/B2-BoNT/X			
Enzymatic substrate (cleavage site)	VAMP1, 2, 3, 4, 5 Ypkt6 (RA)	VAMP2 (WW)	VAMP2 (DL) SNAP25, 23 (KD) syntaxin (MD)	
Neurotoxin-producing bacteria	*C. botulinum* strain 111 group I	*Weisella oryzae*	*Enterococcus faecium*	*Chryseobacterium piperi*

The putative novel BoNTs have not been reported to be responsible for human or animal botulism.

**Table 3:** Binding affinity to receptor of Botulinum neurotoxins (BoNT) and representative potent lethal bacterial toxins.

Toxin	Neuronal Membrane/Receptors	K_d_ Affinity	Reference
BoNT/A	SV2C, neurons	0.46 nM	[58]
BoNT/B	Rat synaptotagmin/GT1b, rat brain synaptosomes	≈0.4 nM	[59]
BoNT/B	Mouse synaptotagmin II Human synatotagmin II	130 nM >20 μM	[60]
Diphtheria toxin (DT)	Heparin Binding-EGF	1.3 nM	[61]
Diphtheria toxin	LCH cells (L cells expressing DT receptor)	0.56 nM	[62]
Protective antigen *Bacillus anthrax* toxin	Capillary morphogenesis protein 2 (CMG2)	0.17 nM	[63]
	Anthrax toxin receptor/tumor endothelial marker 8 (ATR/TEM8)	130 nM	[64]
*Clostridium perfringens* epsilon toxin	Rat brain synaptosome	2.5 nM	[65]
*Clostridium sordellii* lethal toxin	Porcine brain phosphatidyl serine	140 nM	[66]
